# Hepatitis C Direct-Acting Antivirals in the Immunosuppressed Host: Mechanisms, Interactions, and Clinical Outcomes

**DOI:** 10.3390/v17111422

**Published:** 2025-10-26

**Authors:** Hoor AlKaabi, Siham AlSinani, Mohamed El-Kassas, Khalid A. Alswat, Khalid M. AlNaamani

**Affiliations:** 1 Department of Medicine, Division of Internal Medicine, The Medical City for Military and Security Services, Muscat 111, Oman; 2 Department of Child Health, Sultan Qaboos University Hospital, University Medical City, Muscat 123, Oman; 3 Department of Child Health, Sultan Qaboos University Hospital, Sultan Qaboos University, Muscat 123, Oman; 4 Endemic Medicine Department, Faculty of Medicine, Helwan University, Cairo11795, Egypt; 5 Applied Science Research Center, Applied Science Private University, Amman 11931, Jordan; 6 Steatotic Liver Diseases Study Foundation in the Middle East and North Africa (SLMENA), Cairo 11795, Egypt; 7 Liver Disease Research Center, Department of Medicine, College of Medicine, King Saud University, Riyadh 11461, Saudi Arabia; 8 Department of Medicine, Division of Gastroenterology and Hepatology, The Medical City for Military and Security Services, Muscat 111, Oman

**Keywords:** HCV, DAAs, immunosuppressants, biological agents, DDIs, SVR, organ transplant

## Abstract

Direct-acting antivirals (DAAs) have transformed *hepatitis C virus* (HCV) management, offering high cure rates, favorable safety, and simplified regimens. Management in immunosuppressed patients remains challenging due to drug–drug interactions (DDIs). The objective of this review is to summarize clinical outcomes, safety, and pharmacologic considerations of DAA therapy in immunosuppressed patients, including solid organ transplant recipients and those on biological agents. We reviewed clinical studies, pharmacologic databases, and guidelines to characterize DAA classes, mechanisms, and relevant DDIs in immunosuppressed *HCV* patients. In transplant recipients, DAAs achieved sustained virological response (SVR) > 90% with minimal graft rejection. Safety profiles were favorable, and immunosuppressant dose adjustments were rarely needed. DDIs, particularly with calcineurin inhibitors (tacrolimus, cyclosporine), require careful monitoring due to variable trough-level effects. Evidence also supports the efficacy and safety of DAAs in patients on biological agents, without compromising SVR. Pharmacokinetic data indicate DAAs maintain antiviral activity across *HCV* genotypes in the presence of immunosuppressants, though mTOR inhibitors may alter efficacy in certain *HCV* genotypes. DAAs are highly effective and safe in immunosuppressed patients, achieving high SVR rates and potential graft survival benefits. Prospective studies are needed to assess DAA therapy in patients receiving biological agents and to optimize co-administration strategies with immunosuppressive agents.

## 1. Introduction

*Hepatitis C virus* (HCV) infection is a significant global contributor to chronic liver disease [1]. However, the advent of highly effective direct-acting antiviral agents (DAAs) has markedly transformed the management of *HCV*, offering cure rates exceeding 90% even in historically difficult-to-treat populations [2]. The widespread adoption of DAAs has facilitated the development of simplified treatment protocols characterized by high sustained virological response (SVR) rates and favorable safety profiles [3]. Consequently, the prescription and administration of DAAs have extended well beyond hepatology, now involving a broad spectrum of healthcare providers, including gastroenterologists, infectious disease specialists, internists, and primary care physicians [4].

With the expansion of *HCV* management beyond hepatology, it is essential to emphasize that a basic understanding of liver function, drug pharmacodynamics, and patient-specific considerations remains important for safe and effective treatment [5]. Knowledge of the mechanisms of action of DAAs, their hepatic metabolism, and potential drug–drug interactions (DDIs), especially in patients with comorbid conditions or polypharmacy, is important for optimizing therapeutic outcomes [6].

Treating *HCV* infection in patients receiving immunosuppressive therapy presents unique clinical challenges. This population included individuals requiring long-term immunosuppression, such as solid organ transplant recipients and those with autoimmune diseases, who are often managed with complex medication regimens [7].

## 2. Overview of DAAs

**Figure 1:**
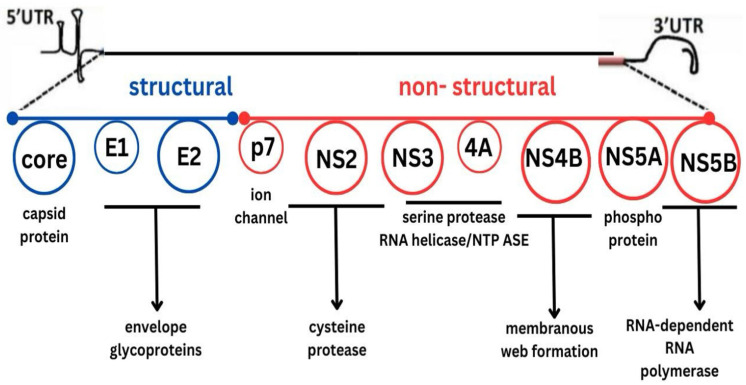
Hepatitis C virus genome. Abbreviations: 5′UTR—5′ untranslated region; 3′UTR—3′ untranslated region; E1—envelope glycoprotein 1; E2—envelope glycoprotein 2; p7—ion channel protein; NS—non-structural.

Before 2011, the standard treatment for *HCV* infection consisted of a combination of pegylated interferon (Peg-IFN) and ribavirin. This regimen is poorly tolerated, associated with numerous adverse effects, and carries a risk of graft rejection in solid organ transplant recipients [8]. Over the past decade, however, DAAs have become central to *HCV* therapy owing to their superior efficacy, improved tolerability, and simplified dosing regimens [3,5]. These agents specifically target the non-structural proteins of the *HCV* genome that are essential for viral replication, thereby enabling rapid viral suppression and high SVR rates (Figure 1) [9].

## 3. Classes of DAAs and Their Mechanisms of Action

DAAs are categorized based on their molecular targets in the *HCV* replication cycle. Their mechanisms of action and clinical applications are summarized in Table 1.

### 3.1. Non-Structural 3/4A (NS3/4A) Protease Inhibitors (PIs)

*NS3/4A* protease inhibitors were the first class of DAAs introduced for clinical use in 2011. These agents inhibit *NS3/4A* serine protease, an enzyme required for the post-translational processing of *HCV* polyprotein, and for viral replication within hepatocytes [10].

First-generation PIs, including telaprevir and boceprevir, have been approved for the treatment of *HCV* genotype 1 and represent a significant advancement in therapy. Administered in combination with Peg-IFN and ribavirin as a triple therapy regimen, these agents improved SVR rates from approximately 40–50% (with Peg-IFN and ribavirin alone) to approximately 70–75% in treatment-naïve genotype 1 patients [11].

Despite their enhanced antiviral efficacy, first-generation PIs have several drawbacks, including complex dosing schedules, significant side effects, extended treatment durations, continued requirement for injectable Peg-IFN, and a low barrier to resistance. Moreover, they exhibited limited activity against non-genotype 1 strains [12].

Second-generation *NS3/4A* PIs, including simeprevir, grazoprevir, glecaprevir, and voxilaprevir, have been developed to address these limitations. These agents demonstrated broader genotype coverage and improved SVR rates and were incorporated into oral regimens in combination with other DAA classes, such as non-structural 5A (*NS5A*) inhibitors (e.g., elbasvir, pibrentasvir, velpatasvir) [13,14]. These advancements have enabled shorter treatment durations (8–12 weeks), better tolerability, and higher efficacy in difficult-to-treat populations, including those with decompensated cirrhosis, renal impairment, post-transplantation status, and prior treatment failure with DAAs [15].

### 3.2. NS5A Inhibitors

*NS5A* inhibitors constitute the second major class of DAAs and were introduced in 2013, beginning with the approval of daclatasvir. These agents target the non-structural protein 5A, a multifunctional phosphoprotein critical to *HCV* replication and virion assembly. *NS5A* inhibitors are potent pan-genotypic agents typically used in combination with non-structural 5B (*NS5B*) polymerase inhibitors or *NS3/4A* protease inhibitors to maximize antiviral efficacy. Commonly used *NS5A* inhibitors include ledipasvir, elbasvir, velpatasvir, and pibrentasvir. Their inclusion in treatment regimens has resulted in shorter therapy durations, improved tolerability, and enhanced effectiveness in patients with advanced fibrosis, renal disease, or prior treatment failure [16].

### 3.3. NS5B Polymerase Inhibitors

The third principal class of DAAs is the *NS5B* polymerase inhibitor, which was introduced in 2013 with the approval of sofosbuvir, a first-in-class nucleotide analog inhibitor. These agents inhibit *NS5B* RNA-dependent RNA polymerase, which is an essential enzyme for *HCV* RNA replication [17].

*NS5B* inhibitors are subclassified into the following categories:

- Nucleotide analog inhibitors such as sofosbuvir are the most widely used and clinically important agents.
- Non-nucleoside inhibitors, such as dasabuvir, are less commonly prescribed.

Sofosbuvir revolutionized *HCV* treatment by providing highly effective once-daily oral therapy with excellent tolerability, forming the backbone of many Peg- IFN -free regimens. When used in combination with other DAAs (e.g., *NS5A* inhibitors or *NS3/4A* PIs), sofosbuvir achieves high SVR rates even in difficult-to-treat populations, including patients with decompensated cirrhosis, *human immunodeficiency virus* (HIV) coinfection, post-transplant status, and prior DAA failure [17].

One of the major advantages of sofosbuvir is its pharmacokinetic profile: it is not metabolized by hepatic cytochrome P450 (CYP450) enzymes, thus minimizing the potential for DDIs and making it particularly suitable for patients with advanced liver disease or those on multiple concomitant medications. This favorable safety and efficacy profile has established sofosbuvir as the cornerstone of modern *HCV* therapy [18].

## 4. Overview of Immunosuppressive Therapies

Immunosuppressive agents are essential for the management of patients undergoing solid organ transplantation, those with autoimmune diseases, inflammatory bowel diseases (IBD), and individuals with solid or hematological malignancies. These medications are critical for preventing graft rejection and for modulating aberrant immune responses [19]. In recent years, the introduction of biological agents, including monoclonal antibodies and fusion proteins targeting specific immune pathways, such as tumor necrosis factor-alpha (TNF-α) (e.g., infliximab and adalimumab), interleukin-6 (e.g., tocilizumab), B cells (e.g., rituximab), and integrins (e.g., vedolizumab), has significantly advanced the treatment of autoimmune and oncologic diseases. These agents are increasingly employed in complex, multidrug regimens by specialists, including rheumatologists, gastroenterologists, hematologists, and oncologists [20].

The concurrent use of *HCV* DAAs in patients receiving immunosuppressants, particularly biological agents and calcineurin inhibitors (CNI), represents a major clinical challenge due to their potential for DDIs. The highest risk of drug–drug interactions between DAAs and immunosuppressive agents occurs with CNIs and mTOR inhibitors, particularly when used in combination with *NS3/4A* protease inhibitors (e.g., paritaprevir, glecaprevir, and grazoprevir) (Table 2). These interactions arise because both CNIs and mTOR inhibitors are extensively metabolized by the cytochrome 3A4 (CYP3A4) enzyme system and transported by P-glycoprotein (P-gp), which are key pathways also affected by protease inhibitors. *NS3/4A* protease inhibitors can act as strong inhibitors of CYP3A4, resulting in marked increases in the blood concentrations of CNIs and mTOR inhibitors, potentially leading to nephrotoxicity, neurotoxicity, or impaired wound healing [21].

These interactions may lead to increased toxicity of immunosuppressive agents (e.g., nephrotoxicity and neurotoxicity) or reduced antiviral efficacy of DAAs. Moreover, some immunosuppressants and biologics can independently alter hepatic enzyme activity, potentially modifying DAA pharmacokinetics [22]. For example, the co-administration of DAAs with immunomodulators, such as rituximab, necessitates close monitoring due to the risk of *hepatitis B virus* (HBV) reactivation and hepatotoxicity. The Liverpool HEP Drug Interactions Database is a valuable tool for evaluating these interactions and guiding safe co-treatment strategies [23].

## 5. Common Classes of Immunosuppressive and Biologic Agents and Their Interaction with DAAs

### 5.1. Calcineurin Inhibitors (CNIs)

Calcineurin inhibitors, including tacrolimus and cyclosporine, are one of the earliest and most clinically important classes of immunosuppressive agents. Cyclosporine, approved in 1983, significantly improves outcomes in solid organ transplantation by enhancing graft survival. Tacrolimus, a more potent agent with a favorable side effect profile and simpler monitoring, was approved in 1994. Both drugs function by inhibiting the phosphatase activity of calcineurin, thereby blocking interleukin-2 (IL-2) transcription and subsequent T-lymphocyte activation and proliferation [24].

Despite their efficacy, CNIs have a narrow therapeutic window and are associated with multiple side effects, including nephrotoxicity, neurotoxicity, hypertension, and metabolic disturbances. Given their metabolism via CYP3A4 and transport via P-gp, CNIs are highly susceptible to DDIs [25]. Certain DAAs may inhibit or induce these metabolic pathways, leading to altered plasma CNI levels, thereby increasing the risk of toxicity or graft rejection [25]. Close monitoring, dose adjustment, and interdisciplinary coordination are essential when CNIs are co-administered with DAAs.

### 5.2. Mammalian Target of Rapamycin (mTOR) Inhibitors

mTOR inhibitors, including sirolimus and everolimus, constitute another critical class of immunosuppressants. Sirolimus was introduced in the late 1990s, followed by everolimus, which has been used in both transplantation and oncology [26]. These agents inhibit mTOR complex 1 (mTORC1), a key intracellular signaling pathway involved in cellular growth, metabolism, and survival. Unlike CNIs, mTOR inhibitors suppress T cell proliferation by directly blocking the response to IL-2 without affecting IL-2 synthesis [27].

mTOR inhibitors are often used in combination with CNIs to reduce the overall toxicity in solid organ transplant regimens. Similar to CNIs, they are metabolized via CYP3A4 and transported by P-gp, making them susceptible to clinically significant interactions with DAAs.

### 5.3. Antimetabolites

Antimetabolites are among the earliest immunosuppressants introduced for clinical use and continue to play a vital role in transplantation and autoimmune disease management. Azathioprine, first approved in 1961, was followed by the introduction of mycophenolate mofetil (MMF) in the 1990s, which offered improved selectivity and tolerability [28]. Methotrexate, another key antimetabolite, is widely used for the prevention of rheumatoid arthritis (RA), psoriasis, and graft-versus-host disease [29].

These agents exert immunosuppressive effects by inhibiting DNA and RNA synthesis, thereby impairing lymphocyte proliferation.

- Azathioprine is a prodrug that is converted to 6-mercaptopurine (6-MP), which blocks purine nucleotide synthesis in rapidly dividing lymphocytes [28].
- MMF is hydrolyzed to mycophenolic acid (MPA), which selectively inhibits inosine monophosphate dehydrogenase (IMPDH), an enzyme essential for de novo purine synthesis in lymphocytes [28].

Antimetabolites are frequently used in combination with CNIs and corticosteroids to achieve synergistic immunosuppression while minimizing toxicity. They are routinely employed to prevent rejection in kidney, liver, and heart transplants and to treat autoimmune diseases, such as lupus nephritis, IBD, and RA. However, they carry the risks of bone marrow suppression, gastrointestinal side effects (e.g., MMF-related diarrhea), and heightened susceptibility to infections [30]. In addition, azathioprine metabolism is influenced by thiopurine methyltransferase (TPMT) activity, and genetic polymorphisms in TPMT can affect the risk of toxicity. Co-administration of DAAs may alter drug exposure via changes in renal function or gastrointestinal absorption, highlighting the importance of vigilant DDI assessment [31].

### 5.4. Corticosteroids

Corticosteroids are among the oldest and most widely used immunosuppressive agents, and their clinical application dates back to the 1950s, following early studies that demonstrated their potent anti-inflammatory and immunomodulatory effects. Agents, such as prednisone, methylprednisolone, and hydrocortisone, remain integral to the treatment of autoimmune diseases, solid organ transplantation, hematologic malignancies, and inflammatory disorders [32]. Their immunosuppressive action is mediated through multiple mechanisms, including the binding of intracellular glucocorticoid receptors that modulate gene transcription and suppress pro-inflammatory cytokine expression (e.g., IL-1, IL-2, TNF-α, and IL-6) [33]. They also inhibit T cell activation, antigen presentation by dendritic cells and macrophages, neutrophil migration, and other key components of the immune response [34]. Although highly effective, corticosteroids are associated with significant adverse effects, particularly with prolonged or high-dose therapies [34]. While corticosteroids are metabolized by CYP3A4, they generally exhibit fewer direct interactions with *HCV* DAAs than with CNIs or mTOR inhibitors. Nonetheless, chronic or high-dose corticosteroid use may increase the risk of viral reactivation, including *HCV, HBV*, and latent infections, such as tuberculosis [35].

### 5.5. Biological Agents

Biological agents are a more recent class of immunosuppressive therapies introduced into clinical practice in the late 1990s and the early 2000s following advancements in biotechnology and monoclonal antibody development. These agents are categorized on the basis of their molecular targets and mechanisms of action. Table 2 summarizes the major classes of biologics, their targets, and associated clinical applications [36].

Biologic agents are widely used to manage immune-mediated inflammatory diseases, such as RA, IBD, psoriasis, psoriatic arthritis, systemic lupus erythematosus, multiple sclerosis, and certain hematologic malignancies (Table 3). Despite their targeted specificity, they are associated with various adverse effects, including an increased risk of opportunistic infections, potential malignancy, and rare autoimmune phenomena, such as drug-induced lupus or demyelinating disorders [37].

## 6. Metabolism of DAAs

Most DAAs are metabolized via hepatic pathways, particularly through the CYP450 enzyme system, most notably CYP3A4, and are transported by hepatic and intestinal P-gp, which collectively regulate their absorption, distribution, and elimination. Additional transporters such as breast cancer resistance protein (BCRP) and multidrug resistance-associated protein 2 (MRP2) also influence the pharmacokinetics of various DAAs, thereby affecting their plasma concentrations and tissue distribution [38]. These metabolic and transport mechanisms have significant clinical relevance, as they create the potential for DDIs when DAAs are co-administered with agents that modulate CYP enzyme or transporter activities.

Sofosbuvir, a nucleotide analog *NS5B* polymerase inhibitor, differs from most DAAs in its pharmacokinetic profile. It undergoes minimal CYP450 metabolism and is activated via intracellular phosphorylation. This drug is predominantly eliminated by renal excretion in its inactive metabolite form [18].

## 7. DAA Treatment Outcomes in Immunosuppressed Populations

*HCV* infection is associated with an increased risk of mortality, diabetes mellitus, de novo or recurrent glomerulonephritis, sepsis, malignancies, and graft failure in renal transplant recipients [39]. Moreover, under conventional immunosuppressive regimens, renal transplantation may accelerate the progression of chronic hepatitis C in *HCV*-infected immunocompetent individuals. Therefore, histopathological evaluation and initiation of antiviral therapy are recommended prior to solid organ transplantation [40].

Historically, interferon-based therapies have been linked to low SVR rates and a significant risk of graft rejection and loss in solid organ transplant recipients. In a systematic review by Wang et al., which compared interferon (IFN) plus ribavirin with Peg-IFN plus ribavirin, the pooled SVR rates were 24% (95% CI: 20–27%) for IFN-based therapy and 27% (95% CI: 23–31%) for Peg-IFN-based therapy. The discontinuation rates were similarly high: 24% (21–27%) for IFN and 26% (20–32%) for Peg-IFN regimens [40].

In contrast, the introduction of DAAs has dramatically improved treatment outcomes in immunosuppressed patients. Numerous studies have demonstrated that DAA-based regimens achieve SVR rates exceeding 90%, comparable to the outcomes in the general *HCV*-infected population. These high cure rates, combined with a favorable safety profile and minimal rejection risk, have enabled the safe transplantation of organs from *HCV*-infected donors with effective post-transplant antiviral treatment [41,42,43,44].

## 8. Outcomes of HCV Treatment Using DAAs in Solid Organ Transplant Recipients

The majority of available data on DAA efficacy in immunosuppressed populations pertain to liver and kidney transplant recipients, given the historically high burden of *HCV* in these groups and the critical impact of viral eradication on graft and patient outcomes. Conversely, evidence in recipients of other organ transplants, such as the heart, lung, and pancreas, is limited and primarily derived from small retrospective studies [42].

A retrospective Brazilian study by Pacheco et al. included 165 patients (108 kidney and 57 liver transplant recipients), most of whom were infected with either genotype 1 or 3. The patients were treated with sofosbuvir-based regimens in combination with *NS3/4A* protease inhibitors and *NS5A* inhibitors. The study reported an SVR of 89.6%, with adverse effects documented in 36% of participants [43].

Another single-center retrospective study by Mansour et al. analyzed 108 transplant patients, of whom 76% had received liver transplants and 13% had undergone kidney transplantation. Tacrolimus was the predominant immunosuppressant (91%), and the most commonly used *HCV* regimen was simeprevir plus sofosbuvir (33.9%). The study reported an SVR rate of 98% with no statistically significant changes in immunosuppressant dosage or trough levels during treatment. Only one case of graft rejection and five episodes (4.6%) of graft dysfunction occurred during the DAA therapy [45].

High SVR rates are associated with improved graft survival and reduced mortality rates. Gaur et al. conducted a retrospective study of 59 *HCV*-infected kidney transplant recipients treated with DAAs and found significant improvements in both outcomes [46]. Similarly, Akin et al. reported the effectiveness and safety of 12–24 weeks of sofosbuvir/ledipasvir ± ribavirin treatment in liver and kidney transplant recipients [47].

Tacrolimus is the most extensively studied immunosuppressant. Its trough levels tend to fluctuate during and after DAA treatment, necessitating close monitoring to prevent underimmunosuppression or toxicity. In a retrospective study of 71 liver transplant recipients, Bixby et al. observed a significant reduction in tacrolimus levels from baseline to 12 weeks post-treatment despite stable dosing [48]. Similar findings were reported by Raschzok et al., who noted that patients treated with DAA showed a significant reduction in tacrolimus trough levels [49]. These findings underscore the importance of frequent monitoring of liver transplant recipients receiving tacrolimus during and after antiviral treatment.

In the previously mentioned study by Pacheco et al., 36.6% of patients experienced DDIs between DAAs and immunosuppressants, resulting in elevated (45%) or reduced (46.7%) CNI trough levels, particularly within the first four weeks of therapy. These fluctuations necessitated dosage adjustments in 35.1% of kidney transplant recipients and 35% of liver transplant recipients. Notably, three patients (1.8%) discontinued tacrolimus therapy owing to adverse interactions. These rates were higher than those reported in smaller studies, possibly because of the inclusion of ribavirin-containing regimens, which are known to contribute to significant DDIs [43].

## 9. Outcomes of *HCV* Treatment Using DAAs in Patients Receiving Biological Agents

Although data on the interactions between DAAs and biological agents remain limited, several studies have provided preliminary insights into this emerging area. A retrospective study from Milan, Italy, evaluated psoriatic patients receiving biologic therapy who were treated for *HCV* between January 2010 and November 2017. The study found that patients treated with DAAs had significantly lower Dermatology Quality of Life Index (DLQI) and Psoriasis Area Severity Index (PASI) scores 24 weeks post-treatment than those treated with Peg-IFN and ribavirin [50]. These findings suggest that DAAs are not only more effective but also better tolerated, with fewer dermatological side effects.

Two recent investigations have elucidated the complex effects of rituximab on *HCV* dynamics and treatment outcomes. A prospective study from Taiwan by Liao et al. examined the effects of different immunosuppressive regimens on *HCV* viral loads in patients with RA. The study reported that rituximab therapy was associated with a significant increase in *HCV* RNA levels, whereas tofacitinib and adalimumab had no such effect, suggesting that the latter agents were unlikely to interfere with the efficacy of DAA therapy [51]. Complementing these findings, a review by Roccatello et al. demonstrated that the combination of DAAs and rituximab in patients with *HCV*-associated cryoglobulinemic vasculitis resulted in the clearance of both viral infection and vasculitis, providing long-term remission without evidence of *HCV* reactivation [50]. These findings underscore the importance of individualized monitoring when using rituximab in the context of DAA therapy. While rituximab alone may transiently increase viral load, its concomitant use with DAAs appears safe and effective when appropriately managed.

Similarly, a retrospective study by Zhou et al. reported that rituximab monotherapy may increase the *HCV* viral load in patients with *HCV*-associated non-Hodgkin lymphoma (NHL); however, the addition of DAAs significantly reduced viral load levels [52]. These results are supported by a systematic review and meta-analysis by Zhang et al. and earlier data from Peveling-Oberhag et al., both of which demonstrated that achieving SVR is strongly associated with lymphoma regression, with an overall response rate of 73% to antiviral therapy [53,54]. Collectively, these findings suggest that DAAs are effective in managing *HCV*-associated B-cell NHL when used in combination with rituximab.

In the context of IBD, a multicenter retrospective study using the ENEIDA registry (National Study on Inflammatory Bowel Disease Applied to Clinical Practice; Estudio Nacional sobre Enfermedad Inflamatoria Intestinal Aplicado a la Práctica Clínica), a prospective Spanish database maintained by the Spanish Working Group on Crohn’s Disease and Ulcerative Colitis (GETECCU), evaluated the safety and efficacy of DAAs in *HCV*-infected patients with IBD. Of the 25,998 patients, 79 were identified as having HCV infection and received DAA therapy. Among them, 39.2% concurrently received immunomodulators or biologics. The study found a 96.2% SVR rate with no significant changes in IBD activity before and after antiviral treatment [55]. These results align with and reinforce the findings from earlier studies, confirming that DAAs are both effective and safe in patients with IBD receiving immunosuppressive therapy.

Despite accumulating evidence on the interaction profiles of established biologic agents, such as rituximab and TNF-α inhibitors, with DAAs, data on newer biologics, particularly interleukin-23 (IL-23) inhibitors, Janus kinase (JAK) inhibitors (e.g., tofacitinib and upadacitinib), and integrin antagonists, remain limited. These agents are increasingly being used in the management of autoimmune diseases and IBD, which frequently coexist with *HCV* infection. Given their different mechanisms of action and limited dependence on CYP-P450 metabolism, these newer biologics may pose a different risk profile for DDIs. However, the absence of prospective systematic evaluations of their safety and efficacy in *HCV*-infected patients receiving DAAs presents a significant knowledge gap. In addition, the long-term effects of immune modulation on viral clearance durability, relapse rates, and liver-related outcomes are poorly understood in this context. Addressing this knowledge gap will require focused prospective studies that evaluate pharmacokinetic interactions, clinical outcomes, and immunological safety in diverse patient populations. Such research is crucial for optimizing treatment strategies in immunocompromised patients undergoing DAA therapy and for guiding future updates to the clinical practice guidelines.

## 10. DDIs Between DAAs and Conventional Immunosuppressants

Tacrolimus remains the most extensively studied immunosuppressant for patients with solid organ transplants treated with DAAs. The potential for clinically significant DDIs between DAAs, particularly glecaprevir/pibrentasvir, and CNIs has been assessed in three open-label Phase I studies. These studies showed that co-administration of glecaprevir/pibrentasvir resulted in a 45% increase in the area under the curve (AUC) for tacrolimus [56]. The AUC reflects total drug exposure over time. An increase in tacrolimus AUC by 45% following co-administration with glecaprevir/pibrentasvir indicates higher systemic exposure due to a pharmacokinetic interaction, underscoring the need for close therapeutic drug monitoring to avoid toxicity.

For cyclosporine, a 100 mg dose had a minimal impact on glecaprevir/pibrentasvir exposure (AUC increase ≤37%), whereas a higher dose of 400 mg significantly increased DAA exposure; glecaprevir and pibrentasvir AUCs increased by 410% and 93%, respectively. Notably, glecaprevir/pibrentasvir did not substantially alter cyclosporine levels at either dose (≤14% change in AUC), indicating that the interaction primarily affected DAA pharmacokinetics rather than cyclosporine metabolism [56].

A separate study by Frey et al. investigated whether the interaction between immunosuppressants and DAAs varies according to *HCV* genotype. The addition of an mTOR inhibitor to daclatasvir increased the antiviral efficacy by approximately 30% in genotypes 2a, 3a, and 4a (*p* ≤ 0.01). Similar enhancements were observed for sofosbuvir and ledipasvir. Conversely, in genotype 1b, combining daclatasvir with an mTOR inhibitor reduced antiviral efficacy by 30% (*p* ≤ 0.01 vs. daclatasvir alone). Importantly, CNIs did not significantly alter the antiviral effects of DAAs in any of the genotypes studied [57].

## 11. DDIs Between DAAs and Biological Agents

Potential DDIs between DAAs and biologics may occur through shared metabolic pathways involving CYP3A4. However, specific studies investigating these interactions remain limited. One prospective study from Taiwan examined patients with *HCV*-infected RA treated with tofacitinib, the TNF-α inhibitor adalimumab, or rituximab, without concomitant DAAs. The study found a significant increase in *HCV* RNA following rituximab treatment (*p* < 0.05), whereas no significant changes were observed with tofacitinib or adalimumab (*p* > 0.05). These findings suggest that tofacitinib and adalimumab may not interfere with DAA efficacy and are unlikely to influence *HCV* RNA replication [51].

Further evidence from Moretti et al. indicates that the co-administration of DAAs and rituximab is generally well tolerated, with no major adverse events reported. However, the authors emphasized the need for further studies to better define the pharmacodynamic interactions and long-term safety of this combination [58]. Taken together, the current evidence suggests that tofacitinib and adalimumab can be safely combined with DAAs, while rituximab may require closer monitoring owing to its potential effects on HCV replication dynamics.

In addition, grazoprevir and elbasvir, two DAAs metabolized by CYP3A4 and transported by OATP1B1/3, offer an example of possible theoretical metabolic interactions [59]. Etanercept, a TNF-α inhibitor, may enhance the activity of these enzymes and transporters by blocking the inflammation-associated suppression of CYP3A4. This, in turn, could accelerate the clearance of grazoprevir/elbasvir and potentially diminish its antiviral efficacy. Although clinical evidence is lacking, the 2020 EASL guidelines recommend close monitoring or dose adjustment when etanercept is administered concurrently with grazoprevir/elbasvir [60].

## 12. Options for DAA Treatment Failure

Despite the high SVR rates exceeding 95% achieved with current DAAs, a small subset of patients experience treatment failure due to factors such as suboptimal adherence, advanced fibrosis, or baseline NS5A resistance-associated substitutions (RASs). Among these, RASs in the *NS5A* region are the most clinically relevant, as they tend to persist for years due to their high replicative fitness and can negatively influence retreatment outcomes [61]. In contrast, sofosbuvir, an *NS5B* nucleotide inhibitor, possesses a high genetic barrier to resistance and broad pan-genotypic efficacy, making resistance exceptionally rare [62]. Notably, low-level RASs (<15%) generally do not impact DAA treatment outcomes.

The American Association for the Study of Liver Diseases–Infectious Diseases Society of America (AASLD–IDSA) guidelines recommend changing treatment strategies based on prior regimen [15]. For patients who fail a sofosbuvir-containing regimen or elbasvir–grazoprevir, the preferred option is sofosbuvir–velpatasvir–voxilaprevir for 12 weeks, with ribavirin added for those with genotype 3 and cirrhosis. Patients who fail glecaprevir–pibrentasvir may be retreated with either glecaprevir–pibrentasvir plus sofosbuvir and ribavirin for 16 weeks, or sofosbuvir–velpatasvir–voxilaprevir for 12 weeks (with ribavirin if cirrhotic). In rare instances of multiple DAA failures, triple-class combinations incorporating *NS3/4A* protease, *NS5A*, and *NS5B* inhibitors with ribavirin for 16–24 weeks are recommended [15]. The interaction between these rescue regimens and immunosuppression is described earlier.

## 13. Clinical Approach to Managing DAAs in Immunosuppressed Patients

**Figure 2:**
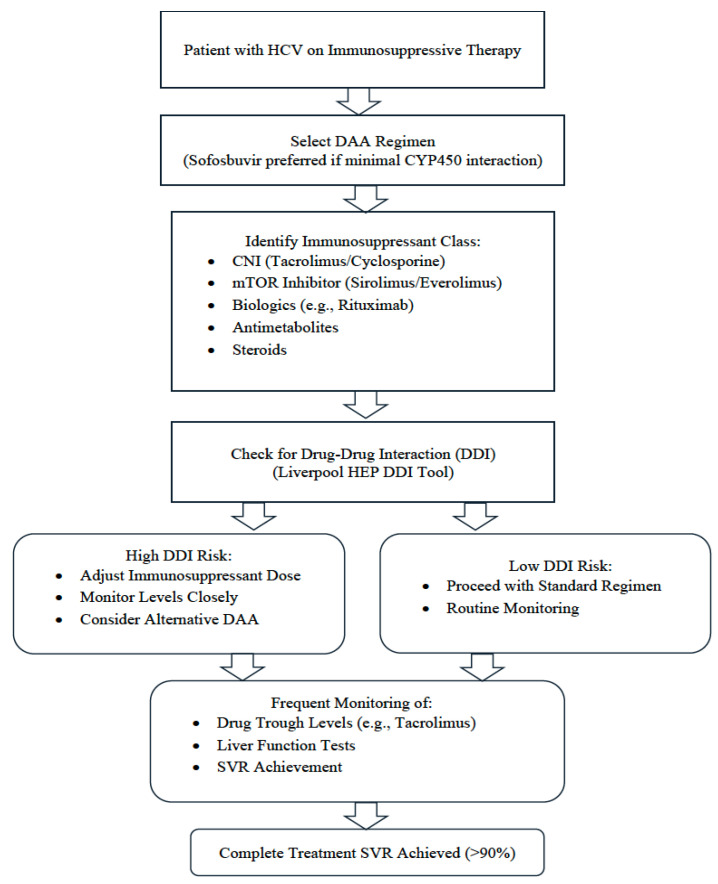
Clinical algorithm for safe use of DAAs in patients on immunosuppressive therapy.

To ensure the safe and effective use of DAAs in immunosuppressed patients, clinicians must carefully evaluate potential DDI, particularly CNIs and mTOR inhibitors, which share metabolic pathways with certain DAAs. The algorithm presented (Figure 2) outlines a practical step-by-step approach to guide clinicians through risk stratification, the selection of appropriate DAA regimens, and monitoring protocols. It emphasizes pretreatment evaluation, careful selection of DAA combinations with minimal potential interaction, frequent monitoring of immunosuppressant levels during therapy, and interdisciplinary coordination, particularly in transplant recipients or patients on biologics. This structured approach aims to optimize SVR while minimizing toxicity and graft-related complications.

## 14. Conclusions

DAAs have revolutionized the treatment landscape of *HCV* infections, including those requiring immunosuppressive therapy, such as solid organ transplant recipients and patients receiving biological agents. The consistently high SVR rates, often exceeding 90%, and favorable safety profiles represent a substantial advancement over interferon-based regimens, which are limited by their low efficacy and higher rates of graft rejection.

Nonetheless, DDIs, particularly CNIs, remain a key clinical consideration because of their variable effects on immunosuppressant trough levels. Current evidence also supports the safety and efficacy of DAAs in patients treated with biological therapies, such as rituximab and tumor necrosis factor-alpha (TNF-α) inhibitors, with no major adverse events or significant compromise in viral clearance. Pharmacokinetic data suggest that DAAs generally retain antiviral activity across *HCV* genotypes when used alongside immunosuppressants, although mechanistic studies have indicated that mTOR inhibitors may modulate DAA efficacy in a genotype-dependent manner.

Despite these promising findings, some important limitations persist in this study. Most available data are derived from liver and kidney transplant recipients, with limited evidence regarding other solid organ transplant populations or those receiving biological therapies. Additionally, the heterogeneity in immunosuppressive protocols, DAA combinations, and study methodologies poses challenges to the generalizability of existing results.

Future large-scale prospective studies are warranted to further elucidate the pharmacological interactions between DAAs and a broader range of immunosuppressive agents, particularly biologics. Such research is critical for developing optimized evidence-based management strategies tailored to these complex and high-risk patient populations.

## Figures and Tables

**Table 1:** Summary of different classes of DAAs, their mechanisms, and clinical uses.

Class	Target	Example Drugs	Main Clinical Use
NS3/4A Protease Inhibitors (PI)	*NS3/4A serine* *protease*	First generation: Telaprevir Boceprevir Second generation: Simeprevir Grazoprevir Glecaprevir Voxilaprevir	First generation limited by side effects and genotype specificity. Second generation effective even in difficult patients (cirrhosis, renal impairment, transplant, prior failure).
NS5A Inhibitors	*NS5A protein* (phosphoprotein involved in replication and assembly)	Daclatasvir, Ledipasvir, Elbasvir, Velpatasvir, Pibrentasvir	Effective in advanced fibrosis, renal disease, and prior treatment failure. Part of most modern *HCV* regimens.
NS5B Polymerase Inhibitors	*NS5B RNA-dependent RNA polymerase*	Sofosbuvir (nucleotide analog), Dasabuvir (non-nucleotide)	Used widely in combination with NS5A inhibitors or PIs. Effective in patients with decompensated cirrhosis, *HIV* coinfection, and transplant.

Abbreviations: NS3/4A, non-structural protein 3/4A serine protease; NS5A, non-structural protein 5A; NS5B, non-structural protein 5B; RNA, Ribonucleic Acid; HCV, hepatitis C virus; HIV, human immunodeficiency virus.

**Table 2:** Potential interaction between immunosuppressant and DAA.

Immunosuppressant Class	Metabolism Pathway	Class of DAA	Interaction Risk	Potential Clinical Impact
CNI	CYP3A4, P-gp	*NS3/4A* Protease Inhibitors	High	High CNI levels associated with nephrotoxicity, and neurotoxicity
mTOR	CYP3A4, P-gp	*NS3/4A* Protease Inhibitors	High	High mTORi levels associated with impaired wound healing, and toxicity
Biologics (Rituximab, TNF-α Inhibitors)	Minimal CYP involvement	Minimal interaction	Low	Generally safe; monitor as needed

Abbreviations: CNI, calcineurin inhibitor; mTOR, mammalian target of rapamycin; DAA, direct-acting antiviral agent; NS3/4A, non-structural Protein 3/4A protease inhibitors; P-gp, P-glycoprotein; CYP, cytochrome; CYP3A4, cytochrome P450 3A4; TNF-α, tumor necrosis factor-alpha.

**Table 3:** Different classes of biological agents, their target molecules, and their clinical uses.

Class	Target Molecules	Example Drugs	Main Clinical Use
IL-1 inhibitors	IL-1	Anakinra	RA, Systemic juvenile idiopathic arthritis
IL-6 inhibitors	IL-6	Tocilizumab, Sarilumab	RA, Giant cell arteritis
IL-17/IL-23 inhibitors	IL-17/IL-23	Secukinumab (IL-17), Ustekinumab (IL-12/23)	Psoriasis, Psoriatic arthritis, Ankylosing spondylitis
TNF-α inhibitors	TNF-α	Infliximab, Adalimumab, Etanercept	RA, IBD, Psoriasis, Ankylosing spondylitis
B-cell depleting agents	CD20	Rituximab	B-cell lymphomas, RA, ANCA-associated vasculitis
T cell co-stimulation modulators	CD80/CD86	Abatacept	RA, Juvenile idiopathic arthritis
JAK inhibitors (small molecules)	Janus kinase pathway	Tofacitinib, Baricitinib	RA, Psoriatic arthritis, Ulcerative colitis
Integrin inhibitors	α4β7 integrin (gut-specific)	Vedolizumab	IBD

Abbreviations: ANCA, anti-neutrophil cytoplasmic antibodies; CD, cluster of differentiation; IBD, inflammatory bowel disease; IL, interleukin; JAK, Janus kinase; RA, rheumatoid arthritis; TNF-α, tumor necrosis factor-alpha.

## Abbreviations

The following abbreviations are used in this manuscript:

DAAs	Direct-acting antivirals
HCV	Hepatitis C Virus
SVR	Sustained virological response
mTOR	Mammalian target of rapamycin
Peg- IFN	Pegylated interferon
NS5A	Non-structural 5A
NS5B	Non-structural 5B
HIV	Human immunodeficiency virus
CYP450	Cytochrome P450
TNF-α	Tumor necrosis factor-alpha
CNIs	Calcineurin inhibitors
CYP3A4	Cytochrome P450 3A4
P-gp	P-glycoprotein
HBV	Hepatitis B virus
IL-2	Interleukin-2
mTORC1	Mammalian target of rapamycin complex 1
MMF	Mycophenolate mofetil
6-MP	6-mercaptopurine
MPA	mycophenolic acid
IMPDH	Inosine monophosphate dehydrogenase
IBD	Inflammatory bowel disease
TPMT	Thiopurine methyltransferase
RA	Rheumatoid arthritis
BCRP	Breast cancer resistance protein
MRP2	Multidrug resistance-associated protein 2
IFN	Interferon
DLQI	Dermatology Quality of Life Index
PASI	Psoriasis Area Severity Index
NHL	Non-Hodgkin lymphoma
AUC	Area under the curve
